# Don’t Blame the System; They’ve Chosen the Wrong One

**DOI:** 10.5334/ijic.4194

**Published:** 2018-07-10

**Authors:** Tony Brauer

**Affiliations:** 1 Community worker/Independent scholar, UK

**Keywords:** integrated care, systems, logic models, markets, communication

## Abstract

While trying to represent patients in the design of integrated care, I have heard the words *system, systemic* and *holism* used frequently. Few of those using the words seem to be aware of the history of systems thinking, or its principles. Health interventions are instead designed using logic modelling, which is aholistic and disintegrative. This concern is illustrated in relation to the UK’s Better Care Fund, which was an attempt to reduce hospital admissions by co-ordinating care. Systems thinking is then used to provide a possible distinction between three operating systems for the UK’s National Health Service (NHS). The first, an ideal market operating system, is inherently fantastic, and doubly so when it is impossible to determine who has contributed what to which outcomes. The accountable professional operating system may re-emerge as the rational option. However, weak analysis can lead to the emergence of a quasi-market operating system, which lacks the capacity to integrate the essential elements of a viable system. The fault lies not with systems thinking, but with the failure to study how viable systems are constructed.

## Systems failure?

I’m 67. The NHS has helped me survive heart attacks and cancer. For over thirty years I’ve used systems thinking, which gives me an opportunity to contribute as a community volunteer. Recently, I’ve been helping to design integrated care for a population of 540,000 in the south of England. I’ve also had direct experience of healthcare in Delhi, north-east Italy and Lesotho. In each location, the essential question has been: who is going to do what, for whom, and why?

Systems thinking is a useful way to seek an answer. If failure is often attributed to systems, it may be because people talk about systems more often than they study systems thinking. This can lead to poor systems designs, while still allowing accountability to be shifted from those who designed the system to the system itself. One familiar factor is reductive linear logic being mistaken for systems design. This is discussed below.

Operating systems will also be discussed. Thinking systemically, we identify different components of a system, but emphasise connections and holism. For holistic functioning, communication between the components must reflect the whole. The communicative sub-systems act as operating systems, dictating performance.

Money is an effective way of communicating some instructions, but not all. The bandwidth is insufficient for the rich qualitative, quantitative and ethical information needed to answer “Who does what, for whom, and why?”. Integrated delivery of holistic health requires a more sophisticated operating system than can be offered by the market alone.

Thus, when market enthusiasts use a truncated version of systems thinking, we are likely to find ourselves with a disintegrative system that fails to address holistic health needs. Then we may hear that there’s been a systems failure, rather than that there’s been a failure to think systemically [1,2].

## Systems and linear logic

By 1972, Stafford Beer had designed a viable system model: that is, a model describing the necessary form of a viable autonomous system [3]. This is a simple narrative variation:

There is stuff. Stuff changes. Now it’s new stuff. The new stuff interacts with its environment. Things change again. We can look at what’s happening and make adjustments. Checking our assumptions often helps.

**Figure 1:**
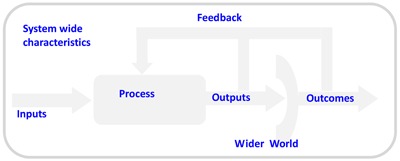
Basic system model [4].

Figure 1 presents this more formally.

Through the description of interconnected sub-systems, even complex narratives can become manageable [5]. The World Health Organisation (WHO) is sympathetic to this view:

Understanding and working with complexity requires a paradigm shift from linear, reductionist approaches to dynamic and holistic approaches that appreciate the multifaceted and interconnected relationships among health system components, as well as the views, interests and power of its different actors and stakeholders [6].

The logic model that the NHS seems to favour is reductive [7,8]. Several elements crucial to a viable system design are omitted.

1. The importance of context (system-wide characteristics) is underestimated or ignored.
2. The design input presupposes linear causality.
3. The wider world is only introduced as an afterthought, by which time genuine critique is crippled by cognitive commitment and confirmation bias.
4. Feedback loops are not structured to respond effectively to unintended outcomes.

**Figure 2:**
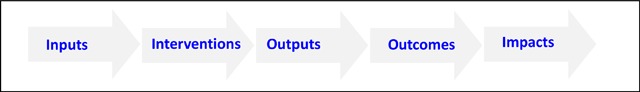
The logic model.

The truncated model is shown in Figure 2.

We are, however, instructed to challenge our assumptions and undertake a reality check [7]. Apply this to the logic model itself, and we may notice that the model doesn’t reflect the complexity of adaptive human systems.

Three levels of systemic complexity can be identified. The first is mechanical, where complexity may arise from the multiplicity of components. Organisms add to the complexity through the obscurity of processes, of which evolutionary adaptation is an example. Complexity in sociocultural systems displays a further qualitative distinction. Adaptation is habitually self-directed, driven by conscious but diverse purposive agency [9].

This sociocultural complexity cannot be satisfactorily addressed by acting as if a complex adaptive human system is an organism or machine. When seeking to model such a system, detail may have to be postponed, but the distinguishing characteristics must be respected, including the high levels of diversity and unpredictability.

In essence, “a good statement about an inherently imprecise concern…. captures that imprecision rather than making a precise statement about something else” [10].

## Evidence

What evidence is there?

The UK’s Better Care Fund (BCF) identified a clear set of objectives: to improve person-centred co-ordinated care in order to reduce admissions to hospital and delayed transfers of care [7]. The design used linear logic, and the prediction was that, in round figures, emergency admissions to hospitals would fall by 100,000, and delayed transfers of care by 300,000. Instead, between 2014 and 2016, there were increases of 100,000 and 200,000 respectively [11].

The logic model neglected the four elements of a viable system identified above. Unrealistic targets and high but unfunded transformation costs were not identified as system characteristics [12]. The design failed to integrate the workforces. The wider world was treated as passive, despite labour market constraints. There were no work streams designed to respond to feedback [11]. The failure of BCF was a failure for logic modelling.

**Figure 3:**
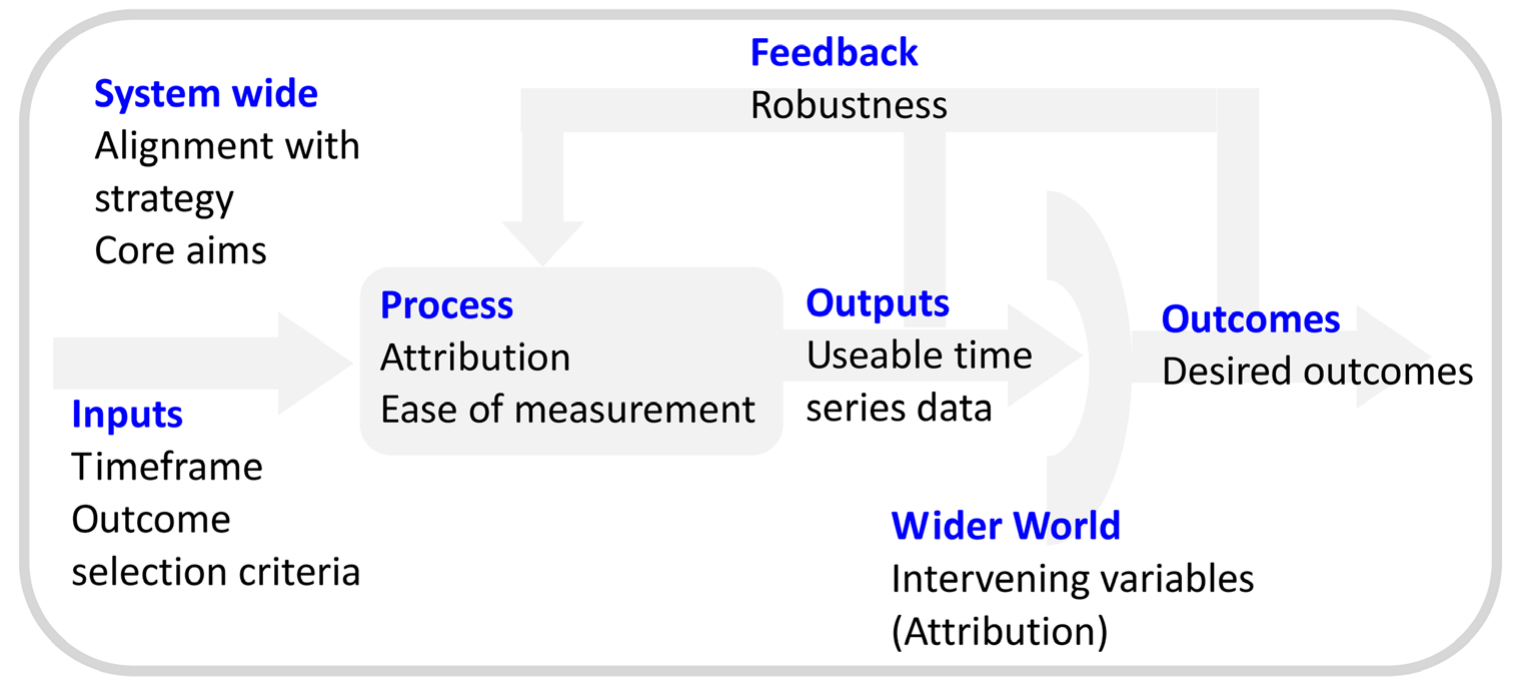
Indicator criteria mapped systemically.

**Figure 4:**
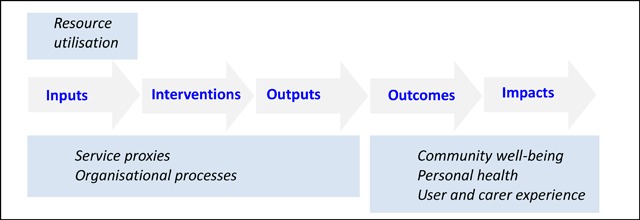
Indicator domains mapped to a logic model.

It is curious that while the criteria for the BCF indicators appear to be systemically viable, the indicators themselves were reduced to a logic model format [7] (Figures 3 and 4).

A very significant omission is feedback, on which learning and adaptation depend. Linear logic models assume a world in which one’s premises are likely to be true, the implications are foreseeable, and the consequences will follow as intended; but logic is about validity rather than real-world interventions [13,14,15].

## A proper use of logic

At first sight, person-centred care appears to be logically immaculate. It is “holistic, *(and)* meets the person’s needs and priorities before those of the system or its professionals…” [16]. Consumer sovereignty in a healthcare market will offer optimal outcomes.

However, placed in context, the principle becomes deeply suspect. If more than one person is seeking care, and if holism implies a concern for their social needs, a concern for others’ needs is entailed [17,18,19]. Holistic healthcare must therefore be people-centred, not person-centred [20]. Furthermore, for intergenerational justice, the system must be sustainable, which implies that staff well-being is critical. Which leg of a three-legged table is least essential? The elements are interconnected and interdependent, and privileging consumer sovereignty contradicts sustainability.

The focus on competitive consumption can also infantilise and marginalise us. If patient concerns are only expressed through “‘I’ statements” [16,21,22], we are excluded from the inescapable decisions about resource allocation, which, in a social healthcare system, are collective not individual [23]. The consumer model also creates intense personal expectations unmatched by resources, and externalises responsibility for health. Systemically, demand will challenge the resilience of staff and the viability of the system. Social solidarity requires a people-centred narrative, in which the interdependence of all stakeholders is recognised.

System models are designed to help us interpret such a world. They are holistic and integrative. Linear logic models, especially when individualistic, are aholistic and disintegrative. Market theory belongs to the individualistic paradigm.

## The market

In the linear logic of the economic imagination, the sum of rationally self-interested decisions is equated to the optimal outcome. A community is entitled to conclude that the optimisation of health is equitably determined by consumer choice, served by the matching of products and prices through an ideal market operating system. Unfortunately, market systems have inherent weaknesses [24]. They are distorted by information asymmetry, monopoly, barriers to market entry and expensive and intensifying inequalities.

In relation to health, markets face an ethical challenge: what if optimisation requires a reasonable opportunity of holistic health for everyone? Unfortunately, an integrated approach to holistic well-being requires a bandwidth beyond the capacity of an ideal market operating system. Failing to align the frame of reference and the operating system results in a dysfunctional hybrid: a quasi-market operating system that cannot carry the signals necessary to the holistic purpose. In effect, inappropriate or contradictory instructions will be given [25].

Apart from the usual market dysfunctions, social provision implies the dominance of a single purchaser, which causes further market failures. Even more fundamentally, outcome metrics for integrated services are intrinsically ambiguous. We simply do not know to which variables in what proportion we should attribute success or failure. A quasi-market operating system intensifies this problem of attribution. Systemic failure is inbuilt.

In the UK, rather than recognise the contradictions into which misapplied linear logic has led them, system designers seem to go into denial. Actors within an integrated care operation are expected to agree in advance how achievement is to be measured and gains/losses are to be shared [8]; but consider patient satisfaction as an indicator. How will we know who has contributed what? Another priority might be staff satisfaction; but this varies with pay and the availability of resources, which are primarily determined by politicians not providers.

The response to these disjunctions was to invoke trust to fill the gaps [8].

## Trust

Trust is justified by the absence of false assumptions. However, service providers are not governed by identical paradigms. Professionals are required by their ethics to pursue the patient’s best interest, while markets only require actors to do what they are contracted to do. The quasi-market is normatively incoherent [26] and lacks objective data to justify the allocation of rewards. How is trust expected to operate in these circumstances?

Meanwhile, competition rules inspired by market theory inhibit the exclusion of those who are not trusted [27,28]. Those who might undermine integration or co-operation could legally be excluded [28], but enforcement is difficult [29]. To predict that others will follow our ethical beliefs rather than their own is a desperate strategy. When profit, loss and organisational survival are functions of ambiguous data, risk-aversion may become the only effective regulator.

So trust is essential, but trustworthiness is not evenly distributed. In the UK, nurses and doctors score ≥90% on a veracity index. NHS managers are just about tolerated (47% – 2015), business leaders less so (36%). Politicians have little credibility (17–19%) [30].

It is unfortunate that those who are least trusted impose the operating system [31: Q373]. At present, efficiency savings seem to be seen as evidence that further cuts can be imposed. Single purchaser power and the threat of competition is used to leverage further cuts, even in those fields where only the professionally committed will bid. Change without investment is tough, going on impossible [32]. Rather than evidence-driven policy, the ideology of public sector austerity appears to have driven the selection of evidence [33,34,35].

An ideal market can function given specific conditions. Those conditions do not prevail in integrated social holistic healthcare systems; and imposing market operating systems on them because market theory looks so good on paper is profoundly irrational.

## In conclusion – the accountable professional

As Adam Smith remarked of the manufacture of pins [36], it can be efficient to break a task into its elements and pay specialists to undertake each element. In such circumstances, an ideal market operating system may allow price and performance to be matched precisely and reliably. There is alignment between the frame of reference, the analytic technique, and the operating system.

However, when the frame of reference is integrated holistic healthcare, that alignment is lost and the market operating system becomes profoundly inadequate. For healthcare of this kind, rich information about roles, incentives and outcomes must permeate a viable system design.

The infinite transaction cost of perfect information is a critical factor. Deliberate ignorance is often a rational response [37], in particular when one has access to trustworthy experts. Such people fit the strong reading of the term *professional*. It is perhaps best to speak of accountable professionalism, in order to emphasise the ethical rather than mercenary dimension of professionalism; and if this option did not exist, it would be necessary to invent it.

However, ignorance is not only a function of the rational allocation of time and the asymmetry of information. Professionals are not omniscient. The pragmatic response is to develop an ethic that promotes behaviours most likely to lead to desirable outcomes [15,38]. Medical codes of ethics suggest that contractual obligations are insufficient. Duty, compassion and accountability must also be incorporated [4,39]. Furthermore, for integrated care, integrated accountability is implicitly required; that is, accountability relating to the performance of the whole system, as well as to individual experiences of it.

That, then is the ground of an accountable professional operating system. It should be capable of supporting a healthcare system that recognises

- culture and cultural change;
- the uncertainty, diversity, incommensurability and complexity of outcomes;
- the need for the iterative challenges of adaptive feedback.

It also recognises that a failure of organisational design is as grievous an error as any medical misdiagnosis. The analytic technique that aligns with this is systems thinking rather than reductive linear logic.

My best hope as a patient is that a systems paradigm will prevail. Market sub-systems may be used as circumstances allow; the maintenance of ambulances, for example, can probably be defined and scheduled effectively and efficiently. However, the best healthcare I’ve received or witnessed in different parts of the world has been driven as much by love and duty as by money. If designers use a reductive paradigm that can’t cope with this, systems will fail. When that happens, don’t blame the system. Hold to account those who designed it.

## Author Information

Tony Brauer has recently been working as a community representative on accountability systems for health services for a half million population in the UK. His background is in systems thinking and community development, including homelessness, arts centres, and environmentalism in the UK, and health services in southern Africa. Along the way he has picked up postgraduate degrees in philosophy, adult education, business and global development. His doctoral thesis discussed the equitable construction of social institutions, and his discussion of three dimensional soft systems analysis – qualitative, quantitative and ethical – has recently been published in the European Journal of Operational Research.
